# The Influence of Cryopreservation on Sperm Morphology and Its Implications in Terms of Fractions of Higher-Quality Sperm

**DOI:** 10.3390/jcm13247562

**Published:** 2024-12-12

**Authors:** Anna Justyna Milewska, Agnieszka Kuczyńska, Michał Pawłowski, Iwo Martynowicz, Sebastian Deluga-Białowarczuk, Piotr Sieczyński, Waldemar Kuczyński, Robert Milewski

**Affiliations:** 1 Department of Biostatistics and Medical Informatics, Medical University of Bialystok, 15-295 Bialystok, Poland; michal.pawlowski@umb.edu.pl (M.P.); robert.milewski@umb.edu.pl (R.M.); 2 Kriobank Infertility Treatment Center, 15-879 Bialystok, Polandiwo.martynowicz@gmail.com (I.M.); sebastiandeluga@wp.pl (S.D.-B.);

**Keywords:** male infertility, cryopreservation, assisted reproductive technologies, semen quality, sperm morphology

## Abstract

**Background/Objectives:** Male infertility is a significant global health issue, comprising approx. 50% of all infertility cases. Semen cryopreservation, a critical component of assisted reproductive technologies (ARTs), is a method commonly used in a wide range of situations, including gonadotoxic treatments such as radiation or chemotherapy, hazardous occupational exposures, and various medical conditions. Although historically viewed as potentially damaging to sperm, recent findings suggest that cryopreservation, when performed with appropriate techniques, may in fact enhance semen quality by improving the proportion of healthy spermatozoa, particularly in terms of their morphological parameters. The aim of this study was to evaluate the impact of cryopreservation on sperm morphology and viability, utilizing advanced morphological assessments pre- and post-freezing. **Methods:** Semen samples were collected from 97 patients undergoing infertility treatment at the KRIOBANK clinic (Białystok, Poland). The semen was liquefied and prepared in the form of slides. Sperm morphology was then assessed using an OLYMPUS BX40 microscope at 60× magnification. **Results:** The findings of the study revealed significant improvements in sperm morphology, with increased percentages of normal sperm and reductions in deformation indices post-thaw. **Conclusions:** The findings indicate that optimized cryopreservation protocols may support the selection of higher-quality sperm, offering valuable benefits for ART applications. These results challenge certain past assumptions regarding the impact of cryopreservation and underscore the need for refined freezing techniques to maintain and potentially enhance semen quality for reproductive use.

## 1. Introduction

Male infertility constitutes a global problem and is believed to be responsible for 40–60% of all infertility cases [1,2], affecting around 2.5–12% of men [1]. As far as its etiology is concerned, male infertility has been classified into pre-testicular, testicular, post-testicular, mixed, and idiopathic [3]. Semen quality assessment is considered a fundamental method for evaluating male fertility [4] and involves both quantitative and qualitative measurements, compared against established reference ranges [5]. The process of selecting the parameters that are responsible for semen quality and thus allow us to predict fertility outcomes with a satisfactory level of certainty is crucial in the context of the assisted reproductive technologies (ARTs) used by fertility clinics.

To standardize these methods, in 1980, the WHO developed a manual, which includes detailed descriptions of semen analysis procedures, reference values, and a nomenclature for disorders. As of 2024, the manual has been revised five times (1987, 1992, 1999, 2010, 2021). It is crucial to note that in the 2010 edition, the reference values for semen parameters were significantly modified (lowered)—compared to previous editions—which led to less frequent diagnoses of male factor infertility, even though the actual number of affected men has in fact increased [6,7,8,9].

Although problems related to male infertility affect a large number of couples worldwide, research has traditionally focused mainly on female fertility. This bias has resulted in an insufficient number of studies, with limited robustness, on male fertility, highlighting the need for further research to test some of the assumptions prevalent in the literature. One topic that requires reevaluation as new data emerge is semen cryopreservation. While long regarded as a procedure that damaged semen, cryopreservation may, in fact, have the opposite effect and may potentially improve semen quality.

In the context of this article—in order to properly clarify the meaning of improved semen quality in terms of cryopreservation—it is important to note that the improvement in question signifies that the ejaculate contains a larger fraction, or percentage, of higher-quality spermatozoa, in comparison to fresh semen. What it does not mean is that a certain number of spermatozoa become biologically improved through freezing. Such an assertion would be both unfounded and impossible to prove, unless only a very small number of spermatozoa are tested that can be compared individually pre- and post-freezing.

The cryopreservation of sperm is an essential practice in assisted reproductive technologies (ARTs). It is commonly used in situations when patients are undergoing gonadotoxic treatments, such as chemotherapy and radiotherapy, as well as in situations when there is a need for fertility preservation due to hazardous occupational exposures or for social reasons [10]. Apart from the aforementioned cases in which cryopreservation can be recommended, there are many more cases in which the procedure is essential. These are listed as clinical indications for depositing male reproductive cells in a sperm bank by The European Association of Urology (EAU) [11] and include surgeries that may impact fertility, progressive deterioration in semen quality due to disease, paraplegia, psychogenic anejaculation, gonadotropin treatment in hypogonadotropic hypogonadism, non-obstructive azoospermia, any situation where sperm is obtained through surgical procedures, and to reduce the risk of infection transmission from donors. According to Directive 2004/23/EC of the European Parliament and Council [12] and Commission Directive 2006/17/EC [13], fresh donor sperm may not be used for non-partner donation. In addition, non-malignant diseases requiring fertility preservation, which include autoimmune diseases, hematopoietic stem cell transplantation, and male genetic disorders, also comprise cases in which cryopreservation is recommended. As can be seen, there are many situations where the cryopreservation of semen is a viable solution, reaching far beyond the cases with which it is traditionally associated, such as malignancies [7].

Sperm cryopreservation is associated with several benefits. It mitigates the need for repeated sperm retrieval procedures, which can be invasive, costly, and uncomfortable for the patient. Therefore, cryopreservation offers both logistical and therapeutic advantages, allowing for synchronized ART cycles and reducing the psychological and physical burden on patients [14]. The literature data also show that the fear that using frozen sperm may negatively affect fertility rates seems to be largely ungrounded. No statistically significant difference has been found between the use of fresh testicular sperm and cryopreserved and thawed sperm in men with azoospermia caused by spermatogenesis dysfunction when assessing clinical pregnancy rates or fertilization outcomes in couples undergoing ICSI [14,15].

Kuczyński et al. [16], citing Tournaye [17], emphasize that cryopreservation can be helpful in eliminating aging or deficient spermatozoa. Based on their own prospective randomized study, the researchers showed that cryopreserved and fresh sperm have at least the same reproductive potential with regard to ICSI. This aspect, i.e., the improvement of semen quality through the selection of higher-quality spermatozoa, is thus the main principle behind the beneficial aspects of cryopreservation.

Sperm cryopreservation has undergone many changes in terms of freezing methods, freezing and thawing rates, and the media used to preserve sperm functionality and DNA integrity [18]. However, it has been linked with a number of problematic issues, mostly related to various ethical issues—concerning sperm banking [19] and the lack of long-term studies on offspring obtained from cryopreserved sperm [20,21]—and the question of its effectiveness. In terms of the effectiveness of cryopreservation, i.e., the resultant semen quality and fertility issues, the main concerns that have been raised are connected with reduced semen parameters such as motility and morphology, DNA damage, and—ultimately—decreased fertilization rates [19,22,23].

A distinction that is absolutely crucial in the context of studies on cryopreservation is that between a proper and improper freezing/thawing process. Researchers have been able to identify the factors that need to be considered in order for cryopreservation to be performed adequately, although there is still no consensus among them as to the ideal technique [10,18,24,25]. The distinction between proper and improper cryopreservation also shifts the focus of discussion from comparing the quality of fresh sperm vs. cryopreserved sperm to fresh sperm vs. properly cryopreserved sperm.

One of the aspects of proper cryopreservation involves determining the optimal sperm selection technique after thawing. It is crucial that we ensure the recovery of the highest-quality sperm with minimal iatrogenic damage. This not only enhances the chances of successful conception but also reduces the risk of genetic damage to the embryo. The results of a study performed by Hungerford et al. [26] showed that electrophoretic sperm selection offers substantial advantages over alternative separation strategies in terms of gamete quality and the time required to complete the procedure. These results confirm both the importance of the separation process and the differences in results achieved with the use of various techniques.

As far as the quality of semen itself is concerned, there are a number of conditions that lead to a decrease in the overall quality of semen. First of all, globozoospermia is a genetic cause of male infertility characterized by a predominant morphological sperm defect that involves a structural abnormality of the sperm head, in which acrosomal pathology coexists with a spherical/round head. No standard reference value has been established for the percentage of sperm with round heads. Electron microscopy studies of such sperm reveal acrosome hypoplasia, sometimes accompanied by a thickened neck, coiled tail, mitochondrial sheath disorganization, axonemal abnormalities, and residual cytoplasm. The use of such sperm for fertilization via ICSI yields a low success rate, i.e., in fertilization (38%), pregnancy (20%), and live birth (14%) [27,28,29,30].

Macrozoospermia, on the other hand, is characterized by up to 100% of sperm in semen having an enlarged, irregularly shaped head, an abnormal acrosome, and multiple tails. In typical cases of macrozoospermia, ultrastructural studies reveal a 3-fold increase in sperm head volume, with an average of 3.6 tails per sperm. Abnormalities in the sperm midpiece are also present. These sperm often exhibit triploidy, tetraploidy, aneuploidy, and increased DNA fragmentation. Currently, fertilization by ICSI is not recommended in such cases. Previous attempts to fertilize oocytes with sperm from men with macrozoospermia mostly resulted in miscarriages due to advanced genetic abnormalities [28,29,31].

Abnormal head–tail attachment in sperm is frequently associated with the absence or abnormal location of the two centrioles that are part of the sperm neck. The most extreme consequence is the independent development of the head and tail, resulting in the presence of pinhead sperm, or sperm without a head (decapitated or acephalic sperm, headless tails, or detached tail defect). In addition to headless sperm, free heads also appear in semen, approx. 30 times fewer than tail fragments. In fertile men, the number of headless sperm can be around 20%, but in cases of male infertility, it is significantly higher, reaching 80–100%. Another form of attachment disorder comprises sperm with a lateral tail attachment. The midpiece of these gametes is positioned at various angles relative to the head (asymmetric midpiece). Sperm may also appear with a gap between the head and midpiece, visible under electron microscopy. These sperm are susceptible to mechanical stress, and spinning or micromanipulation may cause head–tail separation. Patients with numerous sperm showing abnormal head–tail attachment should receive genetic counseling before undergoing ICSI. Success in IVF depends on the extent of the sperm’s morphological and molecular defects [29,32,33].

MMAF (multiple morphological abnormalities of the flagella) syndrome involves various structural anomalies in the sperm tail. These anomalies cause severe asthenozoospermia, in which there may be a complete lack of progressive sperm motility. Light microscopy reveals sperm with bent, abnormally attached, coiled, shortened, or thickened tails, often of irregular shape or entirely missing. The pathomechanism of MMAF syndrome is linked to dysfunction in the genes responsible for the structure and growth of the axoneme, the fibrous sheath structure, flagellum biogenesis, and growth. Due to severe asthenozoospermia, ICSI is recommended. Since MMAF syndrome is frequently genetically determined, there is a risk of passing genetic abnormalities to offspring. The success of ICSI depends on the type and extent of defects, as well as sperm viability in cases of complete immotility [27].

In order to ensure the highest possible semen quality pre-freezing, the appropriate pre-laboratory procedures should be observed [9]. These include the following:

- The patient observing a period of sexual abstinence (number of days since the last ejaculation prior to testing);
- Determining the most beneficial location and method of semen collection, as well as the best sample delivery method and time from semen collection to the start of analysis;
- The choice of the appropriate container;
- Including any comments from the patient upon sample delivery.

In Poland, semen analysis is performed by a laboratory diagnostician or medical laboratory technician who has completed a specialized semen analysis course according to the current recommendations of the National Chamber of Laboratory Diagnosticians (KIDL) and the Polish Andrological Society (PTA) [34].

In addition, the authors’ experience in cryopreservation has led to the following conclusions in terms of various factors connected with sperm freezing that affect sperm motility and morphology after thawing. These factors include the following:

- Genetic defects in sperm that have not been previously considered;
- Incorrect positioning relative to the liquid nitrogen mirror, which can lead to the cooling of the sperm being either too slow or too rapid;
- The immersion of prepared sperm directly into liquid nitrogen without an equilibration step in nitrogen vapors;
- The failure to follow freezing protocols, including freezing times;
- An incorrect medium volume to sperm volume ratio;
- The failure to pre-warm the freezing medium;
- The incorrect method of adding cryoprotectants to the sperm;
- Omitting or shortening the sperm thawing time;
- Too long a delay before starting the freezing procedure (>2 h);
- Too long a time spent transferring frozen sperm to the collection container;
- The failure to maintain a constant temperature for frozen sperm;
- A lack of experience in personnel performing the freezing process, haste, or lack of precision;
- Expired reagents;
- Improper storage and transport conditions for the medium;
- The failure to maintain optimal conditions in the laboratory during sperm thawing.

As far as cryopreservation methods are concerned, slow programmable freezing and liquid nitrogen vapor freezing have been used most widely, though vitrification is gaining attention as an alternative technique with potential benefits in reducing ice crystal formation [24,35]. Slow freezing was considered a beneficial procedure as early as 1990, with research findings pointing out that—despite higher costs and longer processing times—this method was particularly effective in preserving abnormal sperm and therefore superior to the standard rapid freezing method in nitrogen vapors [36].

In sperm cryopreservation, the time of storage in liquid nitrogen does not significantly impact the quality or viability of sperm, as long as the sperm is stored properly at the required low temperatures, such as in liquid nitrogen. What tends to matter more are the freezing and thawing processes themselves, including how quickly the sperm is frozen, whether cryoprotectants are used, and the thawing conditions. According to the calculations by Ashwood-Smith and Grant [37] regarding biological materials stored in liquid nitrogen, damage or chromosome breakage could only occur after approximately 32,000 years. Practically, the storage time of biological materials at temperatures below −130 °C appears to be unlimited [38]. A more recent study (2019) showed that “the long-term cryostorage of semen in a human sperm bank does not affect clinical outcomes. However, cryopreservation longer than 5 years negatively influenced the quality of frozen-thawed donor sperm samples” [39]. Thus, in the context of the above result, variations in freezing times that were inherent in the design of the study conducted by the authors do not appear to affect semen parameters to any considerable degree.

Other studies comparing different cryoprotective media and freezing rates reveal that the choice of cryoprotectant and freezing method significantly influences outcomes such as the DNA integrity, motility, and morphology of thawed sperm [20,40]. Adjustments in protocol, such as pre-freeze sperm selection and the incorporation of antioxidants or osmoprotectants, may further enhance the resilience of cryopreserved sperm, improving clinical outcomes and sperm functionality post-thaw [18,41,42]. In particular, the use of astaxanthin (ASTA) has been shown to improve sperm morphology, although it has no beneficial effect on DNA fragmentation repair [42]. Ghantabpour et al. [43] also reported improved sperm morphology, as well as the protective action of freezing against oxidative stress after freezing with the use of astaxanthin. It has also been pointed out that single-sperm cryopreservation requires a different technique to that of semen cryopreservation, potentially benefiting patients with severe oligospermia or azoospermia [44].

In terms of specific cryopreservation techniques, a crucial distinction in terms of possible cryoinjury is made between slow and fast (or ultra-rapid) freezing, with the literature data suggesting that the former is a safer technique. Hammadeh et al. [45] established that using a programmed slow freezer for sperm freezing and thawing is more effective than rapid freezing with liquid nitrogen vapor as far as preserving sperm chromatin and morphology in semen from both fertile (donor) and subfertile IVF/ICSI patients is concerned. Other studies support this finding, with Morris et al. [46] pointing out that at a rapid freezing rate, sperm cell damage is the result of osmotic imbalance encountered during thawing, rather than the formation of intracellular ice. In addition, slow programmable freezing has been shown to cause fewer morphological changes in sperm compared to freezing sperm in liquid nitrogen vapor [47]. It is worth noting that a successful cryopreservation procedure needs to take into account numerous complex associations between various process parameters and conditions, not only the freezing speed. For instance, Verheyen et al. [48] found freezing–thawing to be most effective when steam freezing was preceded by thawing at 37 °C, and when slower, computer-controlled freezing was combined with thawing at 22 °C, resulting in significant interactions between the freezing method and thawing temperature. For semen samples with high initial quality, both steam and computer-controlled freezing were equally effective in recovering morphologically normal, motile sperm. Other researchers focused on the impact of thawing temperature on semen parameters, showing that thawing at 40 °C resulted in a statistically significant increase in sperm motility recovery compared to thawing at temperatures ranging from 20 °C to 37 °C [49].

The aim of this study was to ascertain that the long-held assertion that cryopreservation may lead to sperm damage—although historically valid—is inaccurate in the light of advances in technologies and methodologies used to cryopreserve semen. In other words, the growing awareness that the quality of semen freezing and thawing processes depends on numerous factors has prompted the need to experimentally test the hypotheses and replicate results of studies that demonstrate the benefits of cryopreservation. Hence, this study aims to show that the chief advantage of properly performed cryopreservation is the selection of high-quality sperm, which is precisely the outcome expected in infertility treatment.

## 2. Materials and Methods

Semen samples (n = 97) were collected via masturbation from patients undergoing infertility treatment at the KRIOBANK clinic (Białystok, Poland).

The method of cryopreservation used in this study was slow-freezing. Before analysis, the semen was liquefied for 30 min at 37 °C. To assess sperm morphology, the Sperm Stain dye (Microptic, Barcelona, Spain) and the Sperm Class Analyzer SCA v 6.6.0.6 software (Microptic, Spain) were used. A 15 µL aliquot of liquefied semen was placed on a glass slide and a smear was prepared. The slide was left to dry for 15 min on a heated plate (37 °C). Staining was performed according to the manufacturer’s instructions for the staining kit, and the slide was again left to dry on the heated plate (37 °C) for 30 min. The same samples were compared before and after the freezing–thawing process. The cryopreservation medium used was GYNEMED G501 SPERM STORE.

**Figure 1:**
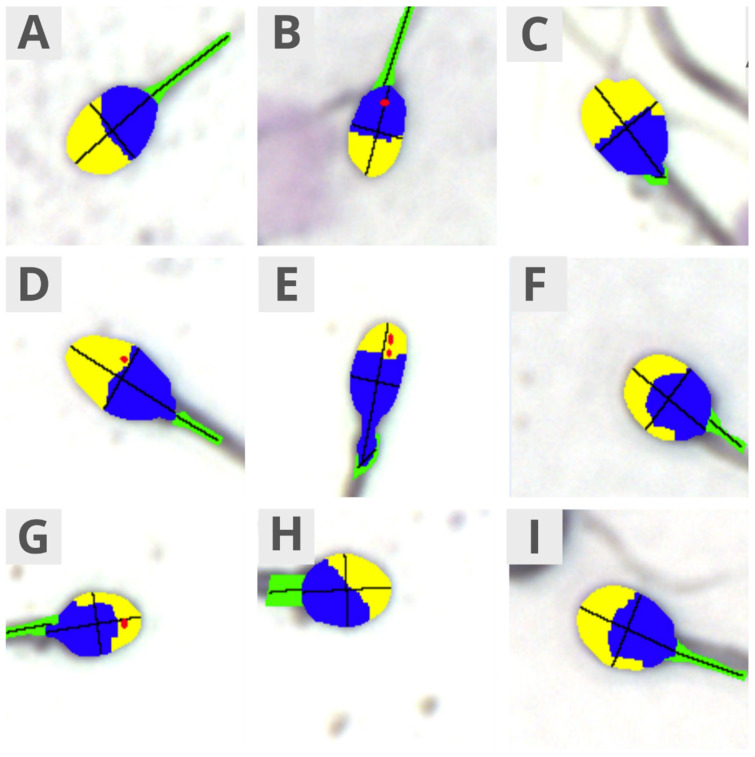
False-color microscopic images taken using the SCA Analyzer representing the following sperm features: head size—normal (**A**), micro (**B**), and macro (**C**); head shape—normal (**A**), conical (**D**), thin (**E**), round (**F**), pear-shaped (**G**), and amorphous (**H**); acrosome—normal (**A**) and abnormal (**E**); midpiece—normal (**A**), abnormal size (**I**), abnormal insertion (**G**), and abnormal angle (**C**); morphometrics—head ellipsoid shape (**E**), head elongation (**E**), head smoothness (**A**), and head regularity (**A**). The colors denote the following parts: yellow, acrosome; blue, head; green, midpiece; red, vacuoles.

Sperm morphology was assessed using an OLYMPUS BX40 microscope at 60× magnification (Tokyo, Japan). The following parameters were evaluated using the SCA software (Figure 1):

- Morphology: normal/abnormal sperm, Teratozoospermia Index, Sperm Deformation Index, and Multiple Anomalies Index;
- Head size: normal, micro, and macro;
- Head shape: normal, conical, thin, round, pear-shaped, and amorphous;
- Acrosome: normal/abnormal;
- Midpiece: normal, abnormal size, abnormal insertion, and abnormal angle;
- Morphometrics: head length, head width, head area, head circumference, ellipsoidal head, elongated head, smooth head, regular head, midpiece width, midpiece area, midpiece angle, and acrosome-to-head ratio.

Quinn’s Advantage Sperm Freezing Medium (SAGE, Ballerup, Denmark) was used for freezing semen. To 0.5 mL of semen, 0.5 mL of medium was added drop by drop at room temperature, while mixing was performed continuously for 30 s. The mixture was then packed into a CBS High Security straw (0.5 mL of the mixture per straw), and the straw was sealed using a sealer. The straws were placed 10 cm above the surface of liquid nitrogen for 20 min and then immersed in liquid nitrogen (−196 °C). After a minimum of 24 h, the straws were placed on a heated plate at 37 °C until the semen sample was thawed (approx. 5 min). The preparation and analyses were performed in the same way as in the case of fresh semen.

### Statistical Analysis

The normality of distribution was tested using the Kolmogorov–Smirnov test with the Lilliefors correction and the Shapiro–Wilk test. A normal distribution was not found for the analyzed quantitative variables. The non-parametric Wilcoxon test was used for comparing two dependent ordinal or quantitative variables. Results were considered statistically significant at *p* < 0.05. The statistical calculations were performed using the Statistica 13.3 software package (TIBCO Software Inc.; Santa Clara, CA, USA).

## 3. Results

A total of 32 semen parameters were analyzed before and after freezing. For 26 parameters, freezing resulted in statistically significant changes in their levels.

**Figure 2:**
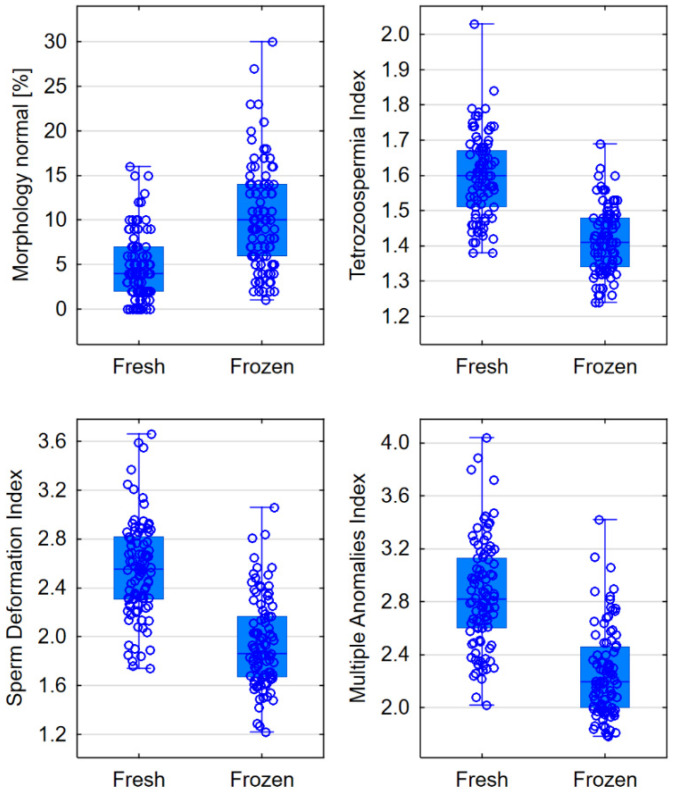
Comparison of sperm morphology in fresh and frozen semen—box plots for parameters that showed statistically significant differences.

After freezing, the percentage of normal sperm averaged 10%, while in fresh semen, the percentage was lower, averaging 4% (*p* < 0.001). Freezing reduced the values of the Deformation Index, Teratozoospermia Index, and Multiple Anomalies Index (*p* < 0.001)—Table 1, Figure 2.

**Figure 3:**
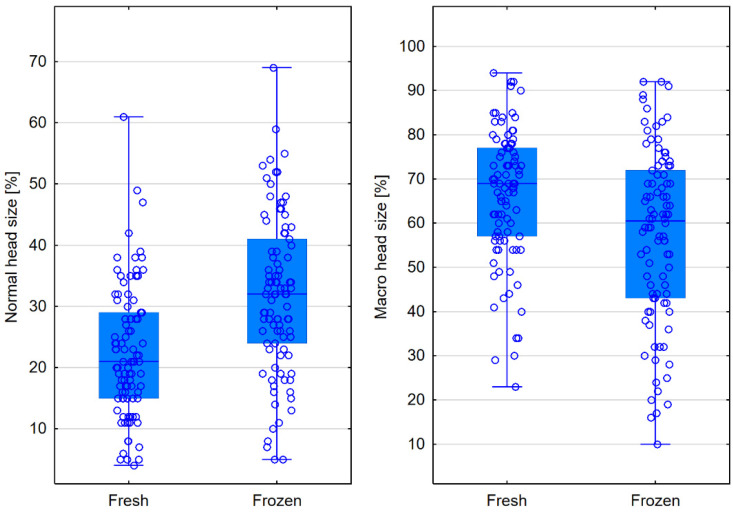
Comparison of head sizes in fresh and frozen semen—box plots for parameters that showed statistically significant differences.

Frozen semen had a higher percentage of sperm with normal-shaped heads compared to fresh semen (32% vs. 21%, *p* < 0.0001). Freezing significantly reduced the percentage of sperm with macrocephalic heads, from 69% to 60.5% (*p* < 0.001)—Table 2, Figure 3.

**Figure 4:**
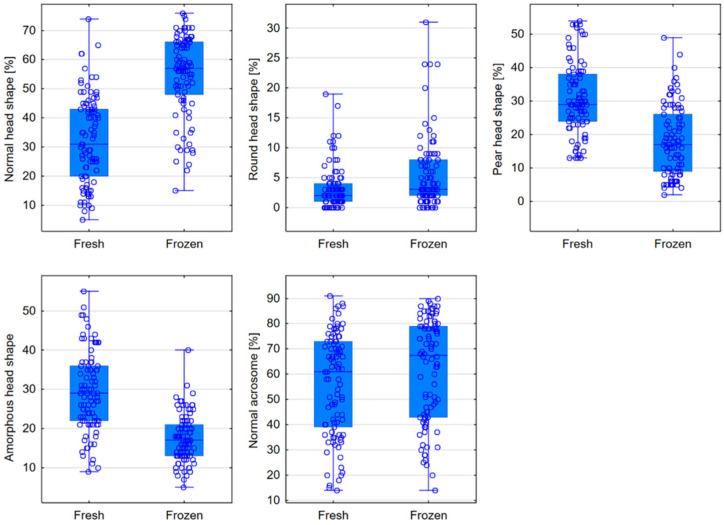
Comparison of head shape and acrosome structure in fresh and frozen semen—box plots for parameters that showed statistically significant differences.

The percentage of sperm with a normal head shape in frozen semen was, on average, 57%, compared to 31% in the case of fresh semen (*p* < 0.0001). Freezing increased the percentage of sperm with round heads, while the percentage of pear-shaped and amorphous heads decreased (*p* < 0.0001). In frozen semen, 67.5% of sperm had a normal acrosome structure, while in fresh semen, only 61% showed a normal appearance (*p* = 0.0009); see Table 3, Figure 4.

**Figure 5:**
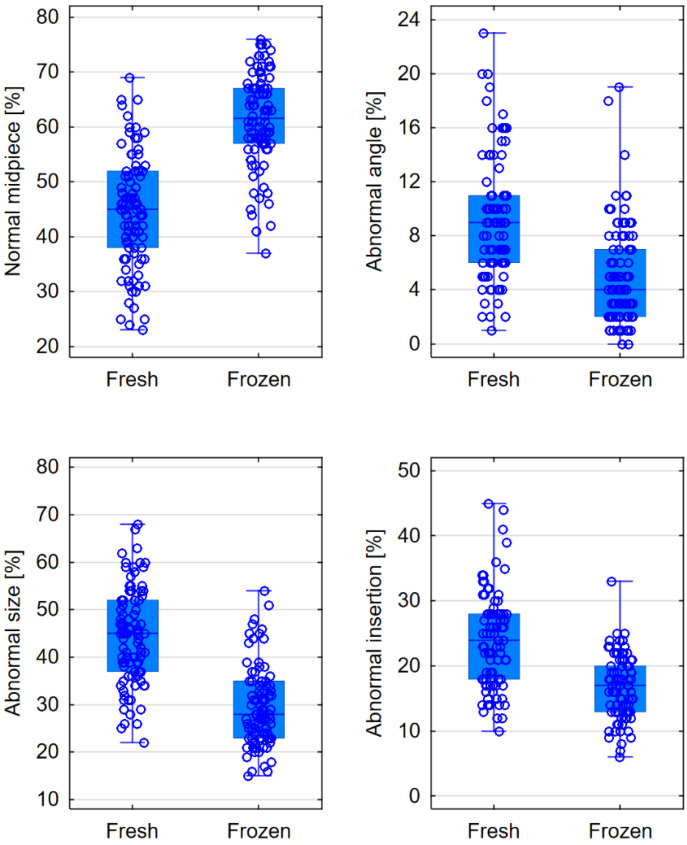
Comparison of the midpiece in fresh and frozen semen—box plots for parameters that showed statistically significant differences.

The evaluation of the midpiece’s appearance revealed that in frozen semen, on average, 61.5% of sperm had a normal midpiece, compared to 45% in the case of fresh semen (*p* < 0.0001). The percentage of sperm with an abnormal midpiece size, abnormal insertion, or abnormal angle was significantly lower in frozen semen (*p* < 0.0001); see Table 4, Figure 5.

**Figure 6:**
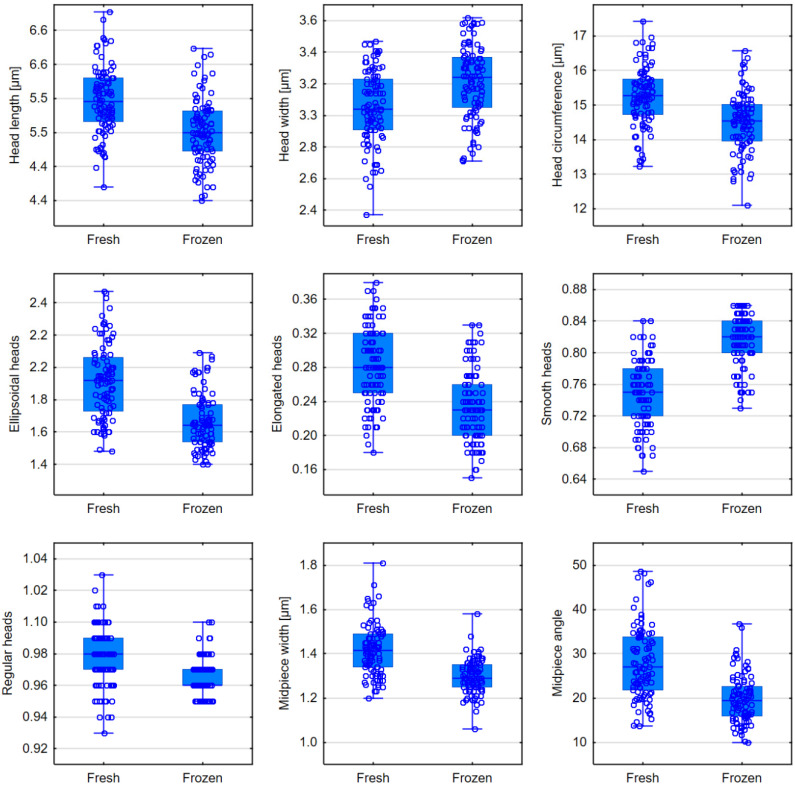
Comparison of morphometric parameters in fresh and frozen semen—box plots for parameters that showed statistically significant differences.

The heads of sperm in frozen semen are statistically significantly shorter than those in fresh semen (5.20 µm vs. 5.57 µm, *p* < 0.0001). The head circumference is also smaller after freezing, but the average head width is greater (*p* < 0.0001). Freezing reduced the percentage of sperm with ellipsoidal, elongated, and regular heads, while the percentage of sperm with smooth heads increased (*p* < 0.0001). The width of the sperm midpiece in frozen semen averages 1.29 µm, compared to 1.42 µm in fresh semen (*p* < 0.0001). The midpiece angle is smaller in frozen semen than in fresh semen (19.36° vs. 27.05°, *p* < 0.0001); see Table 5, Figure 6.

## 4. Discussion

The results of this study show that the cryopreservation of sperm has a beneficial impact on a number of morphological parameters, which challenges the previous assumptions concerning the harmfulness of the procedure. However, in order to interpret the results correctly, they must be viewed in the broader context of various issues connected with the process of freezing and thawing semen, particularly those connected with technological and methodological aspects.

The TZI (Teratozoospermia Index) indicates the average number of defects per abnormal sperm. Hence, it is the ratio of the number of sperm with ≥1 defects in the head, midpiece, or tail or with excess residual cytoplasm to the total number of morphologically abnormal sperm. The maximum possible value of this index is 4. Semen analysis in fertile and subfertile men has revealed a correlation between a sperm’s ability to fertilize an oocyte and its morphology, as expressed by the TZI value [7,50], proposing a TZI threshold value of 2.09 to distinguish fertile men from infertile men.

The SDI (Sperm Deformity Index) reflects the average number of defects per sperm. It is calculated as the sum of all identified head defects, the number of sperm with ≥1 midpiece and tail defects, and the number of sperm with residual cytoplasm, divided by the total number of sperm (both morphologically normal and abnormal). The maximum SDI value is 3. Studies have shown that the SDI for fertile men is between 1.50 and 1.60; for infertile men with low levels of reactive oxygen species (ROS), it is 1.60–1.70, while for infertile men with high ROS levels and leukocytospermia, the SDI value is significantly higher, at 1.90–2.00 [51,52,53,54].

The MAI (Multiple Anomalies Index) measures the presence of multiple abnormalities within a single sperm, indicating the average number of defects per sperm [7]. This is the ratio of the total number of head, midpiece, and tail abnormalities to the number of abnormal sperm. Unlike the TZI and SDI indices, the MAI does not consider sperm with excess residual cytoplasm, and its value remains undefined. Historically, there was considerable skepticism regarding the viability of cryopreserved sperm, despite the fact that the necessity of using it in certain situations [11,14] has been widely accepted. The initial concern stemmed from observations that the freezing process could be destructive to sperm, potentially impacting fertility outcomes when using frozen–thawed sperm compared to fresh sperm [45]. Early studies emphasized the risk of cryoinjury, suggesting that cryopreservation might compromise both the structural integrity and functionality of sperm cells, thus negatively influencing clinical pregnancy rates and embryo quality in ARTs [19], despite results to the contrary, showing that the cryopreservation of testicular sperm is feasible, and the ICSI outcomes using frozen and thawed testicular sperm being similar to those obtained with fresh testicular sperm [55]. Additionally, it was previously believed that cryopreservation led to a significant deterioration in sperm morphology and thus lower fertilization rates [22,23]. It has to be emphasized that the adverse effect of cryopreservation on semen quality was not a universal view in the literature. For instance, Friedler et al. [56] showed that sperm cryopreservation using a simple freezing protocol in patients with obstructive azoospermia was feasible and effective and should be offered to optimize pregnancy outcomes following such procedures. Similarly, another study from the same year (1998) did not show significant differences in the outcomes of using fresh and frozen epididymal sperm for intracytoplasmic sperm injection (ICSI) [57]. On the other hand, Ulug et al. [58], who performed a comparative study, showed that cryopreservation harmed both testicular and ejaculated sperm by causing intracellular ice formation, which ruptured the plasma membrane and allowed oxidative damage to DNA.

Despite the concerns discussed above, advances in freezing techniques, including improved cryoprotectants and controlled freezing protocols, have largely mitigated these early limitations, rendering cryopreserved sperm comparable in efficacy to fresh sperm in many contexts [11,24,59]. It should be noted, however, that cryopreservation can indeed damage sperm through several processes, primarily involving cellular dehydration, oxidative stress [60,61], and osmotic stress [60]. These cumulative effects, known as cryoinjury, underscore the importance of selecting optimal cryoprotectants that can shield sperm cells during the freeze–thaw cycle. Notably, antioxidants have been investigated as supplementary agents to counteract oxidative damage, though the ideal combination remains to be established [18].

Another aspect that plays a crucial role in the success or failure of ART procedures is the fact that sperm selection prior to freezing improves the quality of the cryopreserved semen sample immediately before insemination [41]. This aspect reflects a broader issue connected with semen quality, i.e., the quality vs. quantity dichotomy. As only a single sperm is necessary for a successful conception, procedures that increase the chances that the most viable, high-quality spermatozoa will be used for fertilization also increase the chances of success of the process. It has been shown that sperm selection results in an increase in sperm motility regardless of the separation technique used [18]. Donnelly et al. [62] suggest that isolating the subpopulation of sperm with the highest motility and DNA integrity and freezing them in seminal plasma may be the optimal approach for obtaining high-quality sperm. The results of a study performed by Spanò et al. [63] showed that, post-selection, sperm comprise a subpopulation that is characterized by an overall improvement in morphological parameters and kinetic properties. This fraction also demonstrated improved chromatin structure characteristics, indicating optimal fertilizing potential. This result emphasizes that the process of selection is the key feature of cryopreservation that influences the overall quality of semen post-thawing.

Another aspect that needs to be considered in view of the quality of semen post-freezing is sperm preparation. Palomar Rios et al. [64] suggest that freezing sperm prior to swim-up selection should be considered to achieve better outcomes after thawing, especially in patients presenting with a poor baseline sperm profile.

The results of the present study suggest that freezing may enhance certain sperm parameters, potentially improving the quality of samples for assisted reproductive techniques. The findings show improved semen morphology parameters after cryopreservation with respect to the percentages of morphologically normal sperm before and after the freezing–thawing process. This enhancement may be due to the fact that abnormal sperm were unable to survive the stress and damage caused by the cryopreservation methods, resulting in the loss of many of these abnormal sperm. The presence of primarily healthy and slightly abnormal sperm after thawing could account for the improvement of the parameters that are expressed as fractions/percentages observed in this study. The proportion of morphologically normal sperm post-freezing increased from 4% in fresh samples to 10%. Similarly, a reduction in anomaly indices was observed, with indices describing deformation, teratozoospermia, and multiple anomalies being significantly lower after freezing. In addition, frozen sperm samples showed a higher percentage of normally shaped heads (32% vs. 21%) and correct tail insertions and sizes, with a reduction in macro head sizes and abnormal insertions. These results are in line with the literature data [14] that show that cryopreservation—when performed properly—does not lead to the deterioration of semen quality but may in fact improve it by increasing the fractions of higher-quality spermatozoa in a sample.

The aforementioned result corroborates the findings of a prospective randomized study performed by Kuczyński et al. [16], who investigated fertilization and pregnancy outcomes using fresh versus cryopreserved ejaculated sperm in ICSI. The results demonstrated no significant difference in fertilization and pregnancy rates, suggesting that cryopreservation does not adversely affect reproductive potential. Interestingly, cryopreserved sperm achieved a higher rate of ongoing pregnancies compared to fresh sperm, indicating that cryopreservation may serve as an effective method for selecting viable sperm with greater resilience. These findings reinforce the idea that cryopreservation can be a valuable procedure, especially for patients with compromised semen quality, by enabling the identification and selection of sperm that are more likely to withstand the stresses of ART procedures.

Sperm cryopreservation remains a pivotal technique in reproductive medicine, despite the inherent challenges associated with cryoinjury. The evolution of cryoprotectants, freezing protocols, and sperm selection techniques continues to improve post-thaw semen quality, making cryopreserved sperm a viable option in ARTs and fertility preservation. Future research should aim to refine cryopreservation techniques further, to enhance post-thaw semen parameters and investigate the molecular pathways involved in cryoinjury to optimize outcomes in reproductive health.

## 5. Conclusions

This study demonstrates that cryopreservation, when performed with refined protocols, can improve semen parameters, contradicting the traditional view that freezing compromises semen quality. The post-freezing analyses performed in the study showed significant improvements in sperm morphology. These findings suggest that modern cryopreservation techniques may not only preserve but also enhance the quality of semen, facilitating better outcomes in ARTs. The ability to select higher-quality sperm through freezing presents practical benefits for patients requiring fertility preservation. Both the results of this study and the literature data suggest that instead of avoiding cryopreservation due to the adherence to outdated misconceptions, it is instead crucial that we ensure that the procedure is performed properly. Optimal cryopreservation protocols are thus critical in minimizing cryoinjury and maintaining sperm viability post-thawing. Future studies should further explore cryopreservation protocols and their impact on sperm resilience, aiming to optimize outcomes for male fertility preservation and reproductive health. The authors are currently in the process of designing their next study, focused chiefly on sperm motility. Based on literature reports and our own observations, this direction of study seems to be very promising. When the results of this new research are known, we plan to present a comprehensive overview of the impact of cryopreservation on sperm motility as a separate article.

## 6. Limitations of the Study

This study analyzed a relatively small sample size of 97, limiting the generalizability of the findings. Future research involving larger populations could provide a broader understanding of cryopreservation effects. Additionally, since this study primarily focused on sperm morphology, its results do not allow us to make direct inferences in relation to the impact of cryopreservation on other semen parameters that are important in terms of infertility treatment, in particular, sperm motility.

## Figures and Tables

**Table 1:** Comparison of semen morphology in fresh and frozen semen. Results are presented as medians with the 25th and 75th percentiles.

Morphology	Fresh Semen	Frozen Semen	*p* Level
Normal [%]	4 (2–7)	10 (6–14)	<0.0001
Teratozoospermia Index (TZI)	1.60 (1.51–1.67)	1.41 (1.34–1.48)	<0.0001
Sperm Deformation Index (SDI)	2.56 (2.31–2.82)	1.86 (1.67–2.17)	<0.0001
Multiple Anomalies Index (MAI)	2.82 (2.60–3.13)	2.20 (2.00–2.46)	<0.0001

**Table 2:** Comparison of head size in fresh and frozen semen. Results are presented as medians with 25th and 75th percentiles.

Head Size	Fresh Semen	Frozen Semen	*p* Level
Normal [%]	21 (15–29)	32 (24–41)	<0.0001
Micro [%]	8 (5–15)	6.5 (3–15)	0.2694
Macro [%]	69 (57–77)	60.5 (43–72)	<0.0001

**Table 3:** Comparison of head shapes and acrosome structures in fresh and frozen semen. Results are presented as medians with 25th and 75th percentiles.

Head Shape	Fresh Semen	Frozen Semen	*p* Level
Normal [%]	31 (20–43)	57 (48–66)	<0.0001
Conical [%]	0 (0–1)	1 (0–2)	0.0665
Thin [%]	3 (1–5)	2 (0–5)	0.0580
Round [%]	2 (1–4)	3 (2–8)	<0.0001
Pear-shaped [%]	29 (24–38)	17 (9–26)	<0.0001
Amorphous [%]	29 (22–36)	17 (13–21)	<0.0001
Acrosome			
Normal [%]	61 (39–73)	67.5 (43–79)	0.0009

**Table 4:** Comparison of the midpiece in fresh and frozen semen. Results are presented as medians with 25th and 75th percentiles.

Midpiece	Fresh Semen	Frozen Semen	*p* Level
Normal midpiece [%]	45 (38–52)	61.5 (57–67)	<0.0001
Abnormal size [%]	45 (37–52)	28 (23–35)	<0.0001
Abnormal insertion [%]	24 (18–28)	17 (13–20)	<0.0001
Abnormal angle [%]	9 (6–11)	4 (2–7)	<0.0001

**Table 5:** Comparison of morphometric parameters in fresh and frozen semen. Results are presented as medians with 25th and 75th percentiles.

Morphometrics	Fresh Semen	Frozen Semen	*p* Level
Head length [μm]	5.57 (5.33–5.84)	5.20 (4.98–5.45)	<0.0001
Head width [μm]	3.04 (2.91–3.23)	3.24 (3.05–3.37)	<0.0001
Head area [μm^2^]	13.78 (12.89–14.48)	13.72 (12.69–14.54)	0.7831
Head circumference [μm]	15.27 (14.73–15.75)	14.55 (13.96–15.02)	<0.0001
Ellipsoidal heads	1.92 (1.73–2.06)	1.64 (1.54–1.77)	<0.0001
Elongated heads	0.28 (0.25–0.32)	0.23 (0.20–0.26)	<0.0001
Smooth heads	0.75 (0.72–0.78)	0.82 (0.80–0.84)	<0.0001
Regular heads	0.98 (0.97–0.99)	0.96 (0.96–0.97)	<0.0001
Midpiece width [μm]	1.42 (1.34–1.49)	1.29 (1.25–1.35)	<0.0001
Midpiece area [μm^2^]	1.64 (1.52–1.82)	1.69 (1.57–1.79)	0.7777
Midpiece angle [°]	27.05 (21.86–33.85)	19.36 (15.90–22.57)	<0.0001
Acrosome-to-head ratio [%]	53.46 (48.05–69.86)	58.65 (50.41–70.53)	0.0982

## Data Availability

The data presented in this study are available on request from the corresponding author.
